# Large-scale genomic 2D visualization reveals extensive CG-AT skew correlation in bird genomes

**DOI:** 10.1186/1471-2148-7-234

**Published:** 2007-11-23

**Authors:** Xuegong Deng, Ilkka Havukkala, Xuemei Deng

**Affiliations:** 1 State Key Laboratory of Agrobiotechnology & the Key laboratory of Animal Genetics and Breeding of the Ministry of Agriculture, China Agricultural University, Beijing 100094, China; 2 Northeastern University, College of Science, Shenyang, 110004, China; 3 Auckland University of Technology, Knowledge Engineering and Discovery Research Institute, Auckland, New Zealand

## Abstract

**Background:**

Bird genomes have very different compositional structure compared with other warm-blooded animals. The variation in the base skew rules in the vertebrate genomes remains puzzling, but it must relate somehow to large-scale genome evolution. Current research is inclined to relate base skew with mutations and their fixation. Here we wish to explore base skew correlations in bird genomes, to develop methods for displaying and quantifying such correlations at different scales, and to discuss possible explanations for the peculiarities of the bird genomes in skew correlation.

**Results:**

We have developed a method called Base Skew Double Triangle (BSDT) for exhibiting the genome-scale change of AT/CG skew as a two-dimensional square picture, showing base skews at many scales simultaneously in a single image. By this method we found that most chicken chromosomes have high AT/CG skew correlation (symmetry in 2D picture), except for some microchromosomes. No other organisms studied (18 species) show such high skew correlations. This visualized high correlation was validated by three kinds of quantitative calculations with overlapping and non-overlapping windows, all indicating that chicken and birds in general have a special genome structure. Similar features were also found in some of the mammal genomes, but clearly much weaker than in chickens. We presume that the skew correlation feature evolved near the time that birds separated from other vertebrate lineages. When we eliminated the repeat sequences from the genomes, the AT and CG skews correlation increased for some mammal genomes, but were still clearly lower than in chickens.

**Conclusion:**

Our results suggest that BSDT is an expressive visualization method for AT and CG skew and enabled the discovery of the very high skew correlation in bird genomes; this peculiarity is worth further study. Computational analysis indicated that this correlation might be a compositional characteristic, present not only in chickens, but also remained or developed in some mammals during evolution. Special aspects of bird metabolism related to e.g. flight may be the reason why birds evolved or retained the skew correlation. Our analysis also indicated that repetitive DNA sequence elements need to be taken into account in studying the evolution of the correlation between AT and CG skews.

## Background

According to Chargaff's second parity rule, the two complementary nucleotides will have similar frequencies in complete single stranded DNA (A = T, C = G) [1]. However, local violations of this parity rule have been observed in all known organisms and in bacteria [2]. Thus AT skew (A-T)/(A+T) and CG skew (C-G)/(G+C) can vary wildly in local genome scales. The base skews have been found closely related to genome function domains, such as the origin of replication, gene distribution, transcription and replication direction in bacteria [3-5], plants [6] and mammals [7-9]. An intriguing question is, do the two nucleotide skews (for AT and CG) correlate with each other? Actually, the AT and CG skew are often discussed simultaneously [10,11]. Correlations between AT and CG skews in bacterial genomes and in organellar genomes are also mentioned in several reports [12,13]. Touchon et al. found that TA and GC skews in the coding strand for intronic regions (repeat masked) in human are correlated, and the correlation value is around 0.61 [14]. Thus studying the relation between the AT/CG skews is equally important as the variability in the two skews, and both may have evolutionary meaning for genome composition. The correlations between AT skew and CG skew in higher organisms were generally studied in sets of segments in coding regions or noncoding regions, such as introns and some repeat elements which represent selected or neutral mutations, respectively [15]. Such studies have focused more on mutation mechanisms [7,8,15]. However, the skew correlations have seldom been considered in whole genome or chromosome scale.

In earlier research, CG-skews have normally been analyzed by using cumulative skew diagrams [10,15]. Recently, AT and CG skews were analyzed simultaneously by Touchon et al [9], who calculated cumulative total skew as sum of AT and CG skews over 1 kb non-overlapping windows, enabling prediction of replication origins in mammalian genomes. Software for visualization of GC skew for circular bacterial genomes has been developed [16]. Another similar package is Genome Diagram [17], but methods for larger genomes are needed. We developed a new method enabling handy large-scale visualization and analysis of AT and CG skews for whole genomes and large eukaryotic chromosomes, which is very informative. This new method of 2-D color visualization can represent AT skew and CG skew of all segments up to total length or multiple of 1/1024 of the whole genome in a single figure. We call the tool Base Skew Double Triangle (BSDT). After drawing BSDT images of eukaryotic chromosomes, we found that for chicken and other bird chromosomes, the correlations between the two skews show up very clearly and are also quantitatively very high (correlation over 0.95), while such high values are not reached in chromosomes of any other eukaryote species. We then used further two quantitative methods and different window steps to validate these correlations.

Compared to mammals, chicken/bird genomes have more compact structure, with higher gene density, fewer repeat sequences and specific base skew structure [18,19]. Masking out repetitive sequences prior to analysis from all genomes showed that the level of skew correlation is still higher in birds. We think this has some biological and evolutionary meaning, still to be unraveled. From the increase in correlation values after masking repeats, it seems to be a feature related to evolution of the non-repeat component of the genomes. Our 2-D visualization method is considered to be useful in the study and comparison of nucleotide skews at different scales in various genomes in understanding this phenomenon and its evolution in the phylogenetic tree.

## Results

### BSDTs for eukaryote chromosomes

**Figure 1:**
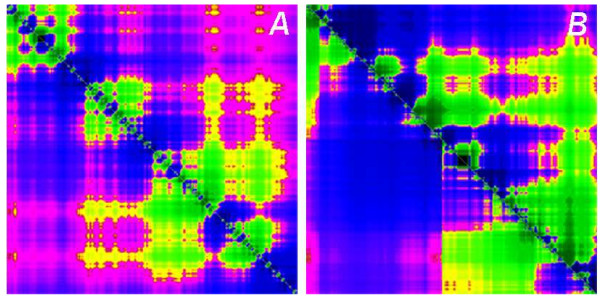
BSDT example of one chromosome of chicken and pufferfish. A is the BSDT of chicken chromosome 5. B is the BSDT of pufferfish chromosome 3. The lower left triangle of each figure represents the AT skew, and the upper right triangle represents the CG skew. The clear correlation of AT and CG skews in chicken chromosome 5 is evident as symmetry across the diagonal. Most chicken chromosomes show such high symmetry in BSDT and all high symmetry BSDTs among eukaryotes belong to chicken.

Our new 2-D color visualization representing AT and CG skews was used, as described in methods, to draw the BSDT pictures (see Methods and Figure 1) for 379 chromosomes of 19 fully sequenced species and two bird species for which extensive genomic scaffold sequences were available. All the BSDT pictures can be found at our website, under link of BSDT Examples[20]. Visual inspection already shows that the bird chromosomes have the most symmetrical BSDTs, even at large scale. The symmetry phenomenon suggests that there is a high correlation between AT and CG skew in chicken and other birds when overlapping windows are used to slide through the whole DNA sequence. The visual symmetry was validated by three quantitative calculations of correlations.

### CNCL – a quantitative index of skew correlation

Constant Number Correlation Level (abbreviated as CNCL) was defined to depict the symmetry degree of BSDTs (see Methods) by calculating correlation of the two base skews at many scales. The degree of the correlation between AT and CG skews was quantitatively validated by the calculation of CNCL using overlapping windows. Two indexes are important for this definition. First, each DNA sequence is divided into 1024 equal segments (1024 for easy fitting on a computer screen); all analysis for CNCL of BSDT pictures is based on this division. Secondly, the step length of overlapping sliding windows is expressed by parameter *β*.

**Figure 2:**
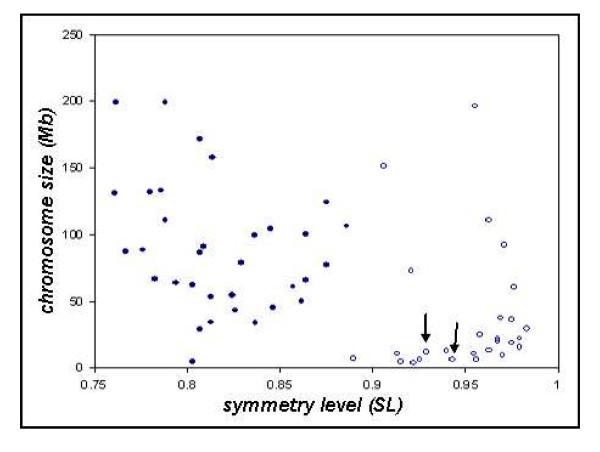
Relation between AT and CG skews correlation level (skew correlation) and chromosome size. Bird data is shown by open symbols, other eukaryotes by closed symbols. Data included: 60 highest CNCL values of all species, cf. Table 2. Left and right arrow: zebrafinch and turkey data points, respectively, other open symbols are all chicken data.

We calculated the CNCL values for all genome data in Table 1 for *β *= 2, 5, 10, 20, 50 by equation (5). Figure 2 shows the CNCL values for 60 datasets with the highest correlations, using *β *= 20. It is remarkable, that chicken chromosomes and the pooled genomic scaffold sequences of two bird species have clearly the highest correlation level by CNCL value ordering. The calculated values can be seen in Table 2.

**Table 1:** Genomic data analyzed, total 19 species, with 379 chromosomes and 2 genomic scaffolds.

Organism	Latin name	Number of chr---size	Data source*
human	Homo sapiens	24---2920 Mb	UCSC (hg18)
chimpanzee	Pan troglodytes	25---2870 Mb	UCSC(panTro2)
dog	Canis familiaris	39---2300 Mb	UCSC(canFam2)
horse	Equus caballus	32---2097 Mb	UCSC (equCab1)
cow	Bos taurus	30---1650 Mb	UCSC(bosTau2)
mouse	Mus musculus	21---2470 Mb	UCSC(mm8)
rat	Rattus norvegicus	22---2560 Mb	UCSC(rn4)
opossum	Monodelphis domestica	10---3420 Mb	UCSC(monDom4)
chicken	Gallus gallus	32---984 Mb	UCSC(galGal3)
turkey	Meleagris gallopavo	28 scaffolds---5.8 Mb	NCBI
zebrafinch	Taeniopygia guttata	92 scaffolds---12.7 Mb	NCBI
stickleback	Gasterosteus aculeatus	24---738 Mb	UCSC (gasAcu1)
medaka	Oryzias latipes	21---408 Mb	UCSC (oryLat1)
pufferfish	Tetraodon nigroviridis	21---211 Mb	UCSC(tetNig1)
zebrafish	Danio rerio	26---1040 Mb	UCSC(danRer4)
mosquito	Anopheles gambiae	6---274 Mb	UCSC(anoGam1)
fruitfly	Drosophila yakuba	6---161 Mb	UCSC(droYak2)
nematode	Caenorhabditis elegans	6---97.5 Mb	UCSC(ce2)
thale cress	Arabidopsis thaliana	5---114 Mb	TAIR(2004)
rice	Oryza sativa	12---364 Mb	BGI
yeast	Saccharomyces cerevisiae	17---11.7 Mb	ENSEMBL(2007)

*UCSC reference to [21], NCBI reference to [25], TAIR reference to [26], BGI reference to [27], and ENSEMBL reference to [28].

**Table 2:** The distribution of all chromosomes/scaffolds by correlation level CNCL at *β *= 20.

Start*	1.00	0.949	0.899	0.849	0.799	0.699	0.599	0.399	0.199	-0.199	-0.399	-0.599	-0.700	Total
End*	0.950	0.900	0.850	0.800	0.700	0.600	0.400	0.200	0.00	0.00	-0.200	-0.400	-0.600	
Chicken	17	7	1	1	1			2			3			32
Turkey		1												1
Zebrafinch		1												1
Dog			5	9	13	7	5							39
Horse			5	2	12	8	4	1						32
Human			2	1	7	7	3	2	1		1			24
Chimp				3	7	4	4	5	1			1		25
Cow				2	3	9	10	5	1					30
Rat					2	5	8	5	2					22
Mouse					2	5	7	6	1					21
Zebrafish					1	5	7	5	4	1	1	2		26
Medaka							3	8	6	3	3	1		24
Rice							1	6	2	2		1		12
Mosquito							1	3	1	1				6
Fruitfly							1	1	3	1				6
Pufferfish							1	1	5	6	4	4		21
Stickleback								3	3	7	3	5		21
Yeast								2	3	5	3	4		17
Opossum								2	1	1	3	3		10
Thale cress								1	3		1			5
Nematode										1	1	1	3	6
Total chr.	17	9	13	18	48	50	55	58	37	28	23	22	3	381
Total random**							34	132	218	235	152	29		800

*Start and end means the range of correlation, the number in table means how many chromosomes/scaffolds have AT/CG skew correlation number between start and end. For example, there are 17 chicken chromosomes having AT/CG skew correlation between 0.95 and 1.00. We can see in this table that birds have higher skew correlation, and no other species reach this level of correlation.

**CNCL distribution of the 800 random sequences. Detailed CNCL data of these random sequences are listed in supplemental materials (file: CNCL for randoms and chromsomes.xls under link of reference) [20].

We also find that, for other vertebrates, a high correlation level is not common, though most vertebrates tend to have higher positive correlations than other lower eukaryotes. In Table 2, we list the distribution of CNCL for all sequenced eukaryotes. Most opossum chromosomes have unexpectedly negative CNCL, as do pufferfish chromosomes. Nematode has a relatively high negative correlation between AT and CG skews. In a control test, 800 = 5 × 16 × 10 CNCLs of random sequences are calculated, with length of 1 Mb, 3 Mb, 10 Mb, 30 Mb, 100 Mb (5 scales) and GC percent of 35%, 36%~50% (16 levels) and 10 repeats for each combination of length scale and GC percent, as listed in Table 2 and details supplied in supplemental file (CNCL for randoms and chromsomes.xls under link of reference in our website [20]). In all the random control sequences, 98.5% of the |CNCL| values are under 0.5, 92.1% |CNCL| < 0.4, 79% |CNCL| < 0.3, 56.6% |CNCL| < 0.2, and 30.4% |CNCL| < 0.1. Compared to the random controls, only warmblooded animal CNCLs can be far from randomness but not for all their chromosomes. Chicken and other birds are the only species in which correlation can reach 0.90 (all chicken macrochromosomes are over 0.95) and the exceptions are all chicken microchromosomes.

Table 2 shows only the case *β *= 20, however, extensive calculations indicated that different settings of *β *do not affect the results significantly. We have:

CNCLβ1chicken-chr. n>CNCLβ2species-chromosomes
 MathType@MTEF@5@5@+=feaafiart1ev1aaatCvAUfKttLearuWrP9MDH5MBPbIqV92AaeXatLxBI9gBaebbnrfifHhDYfgasaacPC6xNi=xI8qiVKYPFjYdHaVhbbf9v8qqaqFr0xc9vqFj0dXdbba91qpepeI8k8fiI+fsY=rqGqVepae9pg0db9vqaiVgFr0xfr=xfr=xc9adbaqaaeGacaGaaiaabeqaaeqabiWaaaGcbaGaem4qamKaemOta4Kaem4qamKaemitaW0aa0baaSqaaGGaciab=j7aInaaBaaameaacqaIXaqmaeqaaaWcbaGaee4yamMaeeiAaGMaeeyAaKMaee4yamMaee4AaSMaeeyzauMaeeOBa4Maeeyla0Iaee4yamMaeeiAaGMaeeOCaiNaeeOla4IaeeiiaaIaeeOBa4gaaOGaeyOpa4Jaem4qamKaemOta4Kaem4qamKaemitaW0aa0baaSqaaiab=j7aInaaBaaameaacqaIYaGmaeqaaaWcbaGaee4CamNaeeiCaaNaeeyzauMaee4yamMaeeyAaKMaeeyzauMaee4CamNaeeyla0Iaee4yamMaeeiAaGMaeeOCaiNaee4Ba8MaeeyBa0Maee4Ba8Maee4CamNaee4Ba8MaeeyBa0MaeeyzauMaee4Camhaaaaa@66D8@

for all conceivable combinations of all species (not bird), and all chromosomes, *β*_1 _= 2, 5, 10, 20, 50, *β*_2 _= 2, 5, 10, 20, 50 and *n *= 1 ~ 14, 18 ~20, 23, 24, 26. Thus our conclusion is that for calculation of CNCL in any scale, chicken chromosomes consistently display distinctly higher correlation compared to other eukaryotes. Here, the segmentation to 1024 parts is only a choice for visual convenience. BSDT is a useful visual observation method, by which we can easily see which genome areas are of interest in terms of skew correlations.

### CWCL – a generalization of CNCL

It could be thought that the skew correlations might not represent a common measurement scale, because the DNA sequences are not of same size, because all are divided into 1024 segments. For this reason, we define a new value CWCL (Constant Window Correlation Level), which does not limit the number of windows to 1024, but limits the window length. In other words, window number for a constant window length replaces 1024. This way we can compare the different genomes in the same scale. The advantage of CWCL is that any two CWCL can be compared in the same scale, though it is not visualized in a common scale square.

We have calculated this standardized scale CWCLβspecies−chr*
 MathType@MTEF@5@5@+=feaafiart1ev1aaatCvAUfKttLearuWrP9MDH5MBPbIqV92AaeXatLxBI9gBaebbnrfifHhDYfgasaacPC6xNi=xH8viVGI8Gi=hEeeu0xXdbba9frFj0xb9qqpG0dXdb9aspeI8k8fiI+fsY=rqGqVepae9pg0db9vqaiVgFr0xfr=xfr=xc9adbaqaaeGacaGaaiaabeqaaeqabiWaaaGcbaGaem4qamKaem4vaCLaem4qamKaemitaW0aa0baaSqaaGGaciab=j7aIbqaaiabdohaZjabdchaWjabdwgaLjabdogaJjabdMgaPjabdwgaLjabdohaZjabgkHiTiabdogaJjabdIgaOjabdkhaYjabcQcaQaaaaaa@4193@ values for all data, *β *= 2, 5, 10, 20, 50 and window-length = 2 k, 10 k. This allows comparisons in same scale between chromosomes and species. Since there are so many possible combinations of beta and window length (WL), all the results cannot be listed here. Table 3 only lists the number of chicken chromosomes that rank highest in correlation for any parameter combination. For example, when *β *= 5 and window-length = 2 k, 25 top CWCL values belong to chicken. The conclusion of Table 3 is quite similar with equation (1), but this calculation is more intuitive, as we are comparing two sequences in the same scale.

**Table 3:** The number of chicken chromosomes that rank highest in correlation for different parameter combinations.

	*β *= 2	*β *= 5	*β *= 10	*β *= 20	*β *= 50
WL = 2 k	24	25	25	25	26
WL = 10 k	26	26	26	27	27

WL is the window length and *β *has the same meaning as in equation 4 for CNCL. The number in table means how many chicken chromosomes have top-ranked AT/CG skew correlation among all data in Table 1 with parameter combination of window length and *β*. For example, when window length is 2 k and *β *= 2, 25 chicken chromosomes have top-ranked correlation values when comparing all chromosomes in Table 1.

### Non-overlapping windows based calculation

**Figure 3:**
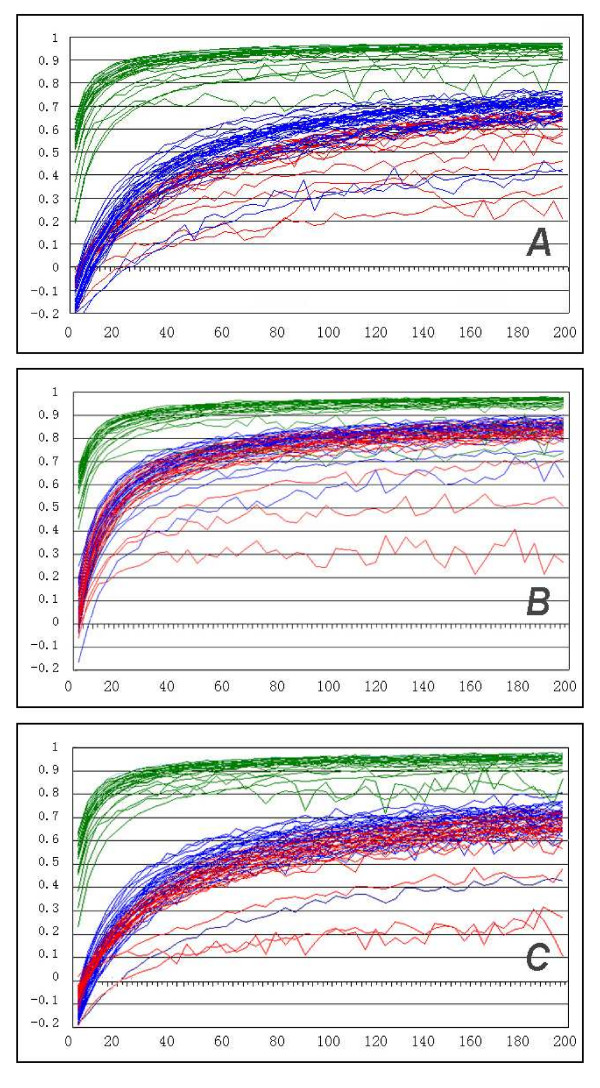
Relation between CG-AT skews correlation (vertical -axis) and window size from 1 kb to 200 kb (horizontal -axis). Each line represents a change of AT/CG correlation of a chromosome with the change of window size. Green: chicken; blue: dog; red: human. A: unmasked genomic data, B: repeats-masked genome data. C: intergenic parts masked genome data.

Intuition and general knowledge suggests that symmetrical phenomena should happen in comparatively large scale, but in small scale, random fluctuations should be dominant. The question is from what scale the chicken/bird genome begins to exhibit its high correlation between skews, or in other words, how its variation changes with the increase of scale. Figure 3A–C shows the relationships between window-sizes and correlation values, using non-overlapping windows. All chromosomes of chicken are drawn in green, for easy comparison with human in red and dog in blue.

It is obvious from Figure 3A that for most chicken chromosomes, the correlation between AT and CG skew rapidly increases to a high level (0.9 at 40 kb and 0.95 at 200 kb or higher), the exceptions being the microchromosomes. For all human and dog chromosomes, none can reach correlation level of 0.8, even at the scale of 200 kb, at the right end of Figure 3A. We can also see that dog has slightly higher correlation level than human, as already seen in the previous analysis of CNCL and CWCL. This seems a species-specific difference. A very interesting phenomenon is that all chicken chromosomes begin at positive correlation level, and all chromosomes of human and dog at negative correlation level at small window sizes. Also, some chromosomes in all species differ significantly from the species specific trend (e.g. lowest curve for human in right panel of Figure 3).

When we eliminated the repeat sequences from the three animal genomes using the standard repeat-masked sequences from the public databases (downloaded from UCSC [21]), the correlations clearly increase in human and dog, and slightly also in chicken, although they already were at a very high level (Figure 3B). This made both dog and human skew correlation levels more similar to each other and closer to that of birds. However, bird chromosome value correlations still stand clearly apart, suggesting that other factors in addition to repeats are responsible for the special characteristic of skew correlation in birds. We also eliminated the intergenic sequences from the three animal genomes using the Genescan gene data in UCSC (see Figure 3C). We only see slight differences between Figure 3C and Figure 3A, but the change is far from that which occurred when masked the repeat sequences. This indicates that the skew correlation in the gene related regions is not significantly different from that in the whole genome.

## Discussion and Conclusion

In this study, we introduced a new visualization method of BSDT to display the changing rules of AT and CG skews in the genome. The visual symmetry in BSDT means high correlation level between AT and CG skews, and high visual symmetry in large scale appeared only in the BSDTs of bird chromosomes. This indicates bird genomes have a very special compositional structure in base skew compared to other species. We validated this estimation by other two quantitative methods, confirming that only bird genomes can reach a high correlation level (0.95). Other species, such as dog, horse and human can also reach a relatively high level (0.8), far from random sequence expectation. However, such high correlations do not appear in rodents and fishes, especially, opossum has an unexpected and unexplained negative correlation. We think this phenomenon is intriguing evolutionarily. Three questions arise: 1) When did this high correlation appear in the vertebrate phylogeny tree? 2) Have birds increased this symmetry due to some evolutionary pressure or have other species lost it (from reptilian ancestors)? 3) Why there are so clear species-specific differences, even within mammals? The high correlation between AT and CG skews is likely to be prevalent in other bird genomes as well, as zebrafinch and turkey genome data showed a very similar picture. The details of the phylogenetic distribution and evolution of this correlation awaits access to more genomic data from further species of birds, mammals and primitive vertebrates, like reptiles.

When the skew correlation is higher than 0.95, we can nearly say that the AT and CG skews change concomitantly. Most chicken larger chromosomes can reach this level at large scale, and for other animals, we can only say they also have tendency to increase in larger scale. The base skew correlation is only a mathematical concept, but when it is far from random state, it must have a biological explanation to maintain it. There are so many vertebrate branches, but why this phenomenon occurred only in the few branches in birds? We have analyzed current genome data, from birds, canines, ungulates, primates, rodents, fishes and marsupials. It is unknown whether the high correlation occurred early, vanishing in marsupials, or whether it occurred reappeared in canines, ungulates, primates etc. at a relatively high level. We need to search for the common ancestor for the high skew correlation state.

It is difficult to judge when birds might have gained such high base skew correlation, either before or after the separation between mammals and birds. New primitive mammal, marsupial, reptile and amphibian data might clarify this. While waiting for such data, we can study further the role of repeat sequences, as well as the history of newly inserted sequences in the evolution process [22,23]. It could be that the high correlation phenomenon occurred very early in the vertebrate phylogeny tree, at least before the separating point of bird and mammal, and then was lost to some extent in mammals. Alternatively, this trait appeared separately in birds and mammals and was strengthened further in the bird branch. We favor the first alternative.

We may need to improve the methods of finding and masking repeat sequences for robust analysis of data from reptiles and common ancestors of mammals and birds. It seems a small number of large-scale recombination events cannot cause an extensive change in skew correlation. Point mutations also cannot be the main reason, since it is a relatively short time for this change in vertebrate phylogeny tree, but mutations maybe a contributing factor. We assume the main reason that most mammals lost the correlation to some extent was numerous random insertions of small sequences with a high difference in AT and CG skew. Such events could involve interspersed repeats, tandem repeats, transposable elements, etc. If we can eliminate such repeats in mammal in a clean manner, mammals may show even higher AT/CG skew correlation, then being more similar to the ancestral genome. These repeats have changed during evolution, and were perhaps not captured well enough by the repeat masking procedure used in the public domain databases, from which we retrieved our repeat masked data.

As we know, avian genomes are commonly smaller and have fewer repetitive elements than other amniotes and that seems a key adaptation for flight [24]. We can also imagine, for the same goal of extremely efficient cell metabolism, birds may adopt a highly organized compositional genome structure, including or reflected in high AT and CG skew correlation. We expect this history to be unraveled soon after more complete genomes are published. It is unknown what possible advantages does the similar AT and CG skew give to the species to manage their genetic information, but it may involve having genes with similar constitution near each other for easier global control mechanisms.

## Methods

### The definition of BSDT

**Figure 4:**
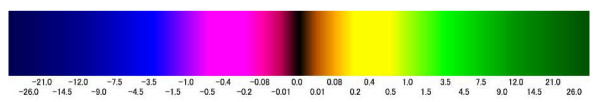
The chromatogram (color scale) drawn using the color function of Table 4. The numbers at the bottom correspond to the skew value, for example 26.0 means when base skew is 26.0*λ*, the color in BSDT is deep green.

The aim of this method is to visualize how the AT and CG skews change and correlate at all scales in a single image for a whole chromosomes. For convenient display on computer screens, we limit the square scale as 1024 × 1024 pixels (choice of 1024 is handy for display on ordinary computer screen, but one can choose any other scale). First, we divide the DNA sequence into 1024 equal size windows *W*^*i*^, all possible continuous segments composed with *W*^*i *^can be denoted as:

Hm,n=∪i=m−1nWi
 MathType@MTEF@5@5@+=feaafiart1ev1aaatCvAUfKttLearuWrP9MDH5MBPbIqV92AaeXatLxBI9gBaebbnrfifHhDYfgasaacPC6xNi=xI8qiVKYPFjYdHaVhbbf9v8qqaqFr0xc9vqFj0dXdbba91qpepeI8k8fiI+fsY=rqGqVepae9pg0db9vqaiVgFr0xfr=xfr=xc9adbaqaaeGacaGaaiaabeqaaeqabiWaaaGcbaGaemisaG0aaSbaaSqaaiabd2gaTjabcYcaSiabd6gaUbqabaGccqGH9aqpdaWeWbqaaiabdEfaxnaaCaaaleqabaGaemyAaKgaaaqaaiabdMgaPjabg2da9iabd2gaTjabgkHiTiabigdaXaqaaiabd6gaUbqdcqWIQisvaaaa@3DAB@

i.e. the segments from the beginning of window *W*^*m*-1 ^to the end of window *W*^*n*^. Then, we denote the base skews of AT and CG of all these segments *H*_*m*,*n *_as Skewm,nAT
 MathType@MTEF@5@5@+=feaafiart1ev1aaatCvAUfKttLearuWrP9MDH5MBPbIqV92AaeXatLxBI9gBaebbnrfifHhDYfgasaacPC6xNi=xH8viVGI8Gi=hEeeu0xXdbba9frFj0xb9qqpG0dXdb9aspeI8k8fiI+fsY=rqGqVepae9pg0db9vqaiVgFr0xfr=xfr=xc9adbaqaaeGacaGaaiaabeqaaeqabiWaaaGcbaGaem4uamLaem4AaSMaemyzauMaem4DaC3aa0baaSqaaiabd2gaTjabcYcaSiabd6gaUbqaaiabdgeabjabdsfaubaaaaa@373C@ and Skewm,nCG
 MathType@MTEF@5@5@+=feaafiart1ev1aaatCvAUfKttLearuWrP9MDH5MBPbIqV92AaeXatLxBI9gBaebbnrfifHhDYfgasaacPC6xNi=xH8viVGI8Gi=hEeeu0xXdbba9frFj0xb9qqpG0dXdb9aspeI8k8fiI+fsY=rqGqVepae9pg0db9vqaiVgFr0xfr=xfr=xc9adbaqaaeGacaGaaiaabeqaaeqabiWaaaGcbaGaem4uamLaem4AaSMaemyzauMaem4DaC3aa0baaSqaaiabd2gaTjabcYcaSiabd6gaUbqaaiabdoeadjabdEeahbaaaaa@3726@, and the whole genome skew denoted as skew denoted as:

*λ *= |(*A *- *T*)/(*A *+ *T*)| + |(*C *- *G*)/(*C *+ *G*))|

in which A, T, C, G are the number of these nucleotides in the whole sequence. Next, we map the two base skews Skewm,nAT
 MathType@MTEF@5@5@+=feaafiart1ev1aaatCvAUfKttLearuWrP9MDH5MBPbIqV92AaeXatLxBI9gBaebbnrfifHhDYfgasaacPC6xNi=xH8viVGI8Gi=hEeeu0xXdbba9frFj0xb9qqpG0dXdb9aspeI8k8fiI+fsY=rqGqVepae9pg0db9vqaiVgFr0xfr=xfr=xc9adbaqaaeGacaGaaiaabeqaaeqabiWaaaGcbaGaem4uamLaem4AaSMaemyzauMaem4DaC3aa0baaSqaaiabd2gaTjabcYcaSiabd6gaUbqaaiabdgeabjabdsfaubaaaaa@373C@ and Skewm,nCG
 MathType@MTEF@5@5@+=feaafiart1ev1aaatCvAUfKttLearuWrP9MDH5MBPbIqV92AaeXatLxBI9gBaebbnrfifHhDYfgasaacPC6xNi=xH8viVGI8Gi=hEeeu0xXdbba9frFj0xb9qqpG0dXdb9aspeI8k8fiI+fsY=rqGqVepae9pg0db9vqaiVgFr0xfr=xfr=xc9adbaqaaeGacaGaaiaabeqaaeqabiWaaaGcbaGaem4uamLaem4AaSMaemyzauMaem4DaC3aa0baaSqaaiabd2gaTjabcYcaSiabd6gaUbqaaiabdoeadjabdEeahbaaaaa@3726@ of H_*m*,*n *_at two symmetrical pixels (m, n) and (n, m) in the square with a color function, in order to get a color square representation for each DNA sequence. This color function is shown in Table 4 and the corresponding color scale in Figure 4. For all points of the matrix we draw (m, n) and (n, m) (at the symmetry position) with *color *(Skewm,nAT
 MathType@MTEF@5@5@+=feaafiart1ev1aaatCvAUfKttLearuWrP9MDH5MBPbIqV92AaeXatLxBI9gBaebbnrfifHhDYfgasaacPC6xNi=xH8viVGI8Gi=hEeeu0xXdbba9frFj0xb9qqpG0dXdb9aspeI8k8fiI+fsY=rqGqVepae9pg0db9vqaiVgFr0xfr=xfr=xc9adbaqaaeGacaGaaiaabeqaaeqabiWaaaGcbaGaem4uamLaem4AaSMaemyzauMaem4DaC3aa0baaSqaaiabd2gaTjabcYcaSiabd6gaUbqaaiabdgeabjabdsfaubaaaaa@373C@) and *color *(Skewm,nCG
 MathType@MTEF@5@5@+=feaafiart1ev1aaatCvAUfKttLearuWrP9MDH5MBPbIqV92AaeXatLxBI9gBaebbnrfifHhDYfgasaacPC6xNi=xH8viVGI8Gi=hEeeu0xXdbba9frFj0xb9qqpG0dXdb9aspeI8k8fiI+fsY=rqGqVepae9pg0db9vqaiVgFr0xfr=xfr=xc9adbaqaaeGacaGaaiaabeqaaeqabiWaaaGcbaGaem4uamLaem4AaSMaemyzauMaem4DaC3aa0baaSqaaiabd2gaTjabcYcaSiabd6gaUbqaaiabdoeadjabdEeahbaaaaa@3726@) for *m *≤ *n *≤ 1024.

**Table 4:** The definitions of color function.

*α *< 0	Color(*α*)	*α *< 0	Color(*α*)	*α *> 0	Color(*α*)	*α *> 0	Color(*α*)
> -26.0*λ*	0,0,80	-3.5*λ*	0,0,228	0.0*λ*	0,0,0	4.5*λ*	0,201,0
-26.0*λ*	0,0,80	-1.5*λ*	0,0,255	0.01*λ*	190,85,0	7.5*λ*	0,174,0
-21.0*λ*	0,0,97	-1.0*λ*	85,0,255	0.08*λ*	255,170,0	9.0*λ*	0,145,0
-14.5*λ*	0,0,113	-0.5*λ*	170,0,255	0.2*λ*	255,255,0	12.0*λ*	0,129,0
-12.0*λ*	0,0,129	-0.2*λ*	255,0,255	0.5*λ*	170,255,0	14.5*λ*	0,113,0
-9.0*λ*	0,0,145	-0.08*λ*	255,0,170	1.0*λ*	85,255,0	21.0*λ*	0,97,0
-7.5*λ*	0,0,174	-0.01*λ*	190,0,85	1.5*λ*	0,255,0	26.0*λ*	0,80,0
-4.5*λ*	0,0,201	-0.0*λ*	0,0,0	3.5*λ*	0.228,0	> 26.0*λ*	0,80,0

The color function Color(*α*) is defined as this table, where *α *is the AT or CG skew and *λ *is |(*A *- *T*)/(*A *+ *T*)| + |(*C *- *G*)/(*C *+ *G*))| (equation 3) for whole genome. Since the *λ *is different for genomes, the color function depends on the value of *λ*. Corresponding color scale is in Figure 4. *Color*(*α*) maps the AT or CG skew *α *to a RGB color. For example if the skew value is -3.5*λ *(top of the third vertical row) the color is (0, 0, 228). The *λ *may be different for different chromosomes.

Using the color scheme in Table 4 we thus get a color square representation for base skews of any DNA sequence, as shown in Figure 1A–B. This square consists of two triangles, separated by the diagonal, the bottom-left for the AT skew and the top-right for CG skew. We call these images Base Skew Double Triangles (BSDT). The structure of these images is explained further below. Since each point in the square represents base skew of a different subsequence, 1,024^2^/2 = 524,288 segments are shown in one triangle. This display with a suitable color scaling highlights very clearly and continuously the changes in AT and CG skews at different scales and in different locations of the DNA sequence.

### What can we see in BSDT?

When observing a BSDT image (see Figure 1), following items are important: 1) Any point pair ((m, n) and (n, m), m < n) in the square represents a sub-sequence H_*m*,*n *_and the color at these pair points represent base skews Skewm,nAT
 MathType@MTEF@5@5@+=feaafiart1ev1aaatCvAUfKttLearuWrP9MDH5MBPbIqV92AaeXatLxBI9gBaebbnrfifHhDYfgasaacPC6xNi=xH8viVGI8Gi=hEeeu0xXdbba9frFj0xb9qqpG0dXdb9aspeI8k8fiI+fsY=rqGqVepae9pg0db9vqaiVgFr0xfr=xfr=xc9adbaqaaeGacaGaaiaabeqaaeqabiWaaaGcbaGaem4uamLaem4AaSMaemyzauMaem4DaC3aa0baaSqaaiabd2gaTjabcYcaSiabd6gaUbqaaiabdgeabjabdsfaubaaaaa@373C@ and Skewm,nCG
 MathType@MTEF@5@5@+=feaafiart1ev1aaatCvAUfKttLearuWrP9MDH5MBPbIqV92AaeXatLxBI9gBaebbnrfifHhDYfgasaacPC6xNi=xH8viVGI8Gi=hEeeu0xXdbba9frFj0xb9qqpG0dXdb9aspeI8k8fiI+fsY=rqGqVepae9pg0db9vqaiVgFr0xfr=xfr=xc9adbaqaaeGacaGaaiaabeqaaeqabiWaaaGcbaGaem4uamLaem4AaSMaemyzauMaem4DaC3aa0baaSqaaiabd2gaTjabcYcaSiabd6gaUbqaaiabdoeadjabdEeahbaaaaa@3726@ the similar colors (in Figure 4) of the two points represent the correlation of two base skews. 2) The skew level of any segment displayed in the BSDT shows strong negative skews as deep blue, changing via black color at no skew to deep green for strong positive skews (see color scale in Figure 4). 3) The closer the points to the diagonal, the smaller the sub-sequence scale it represents. 4) The symmetrical color pattern across the diagonal means correlation of CG skew and AT skew of that genome area. 4) By comparing the color changes along the diagonal direction we can observe local deviations in skews and their correlations. It is noteworthy, that in the corners furthest from the diagonal (= largest genomic scale) the skew is less (color is often pink or yellow, that is, closer to black portion in the middle of the color scale).

### The definition of constant number correlation level of BSDT

The visual observation clearly supports the conclusion that the correlation between the AT skew and CG skew is very high for most large chromosomes of chicken at any scale. However, adequate quantitative analysis of this visual observation (BSDT) is necessary. For this we calculate correlation as follows:

Taking 12 pairs of symmetrical lines parallel to the diagonal in the square,

{m−n=β×k+1m−n=β×k+1
 MathType@MTEF@5@5@+=feaafiart1ev1aaatCvAUfKttLearuWrP9MDH5MBPbIqV92AaeXatLxBI9gBaebbnrfifHhDYfgasaacPC6xNi=xI8qiVKYPFjYdHaVhbbf9v8qqaqFr0xc9vqFj0dXdbba91qpepeI8k8fiI+fsY=rqGqVepae9pg0db9vqaiVgFr0xfr=xfr=xc9adbaqaaeGacaGaaiaabeqaaeqabiWaaaGcbaWaaiqaaeaafaqabeGabaaabaGaemyBa0MaeyOeI0IaemOBa4Maeyypa0dcciGae8NSdiMaey41aqRaem4AaSMaey4kaSIaeGymaedabaGaemyBa0MaeyOeI0IaemOBa4Maeyypa0Jae8NSdiMaey41aqRaem4AaSMaey4kaSIaeGymaedaaaGaay5Eaaaaaa@4492@

**Figure 5:**
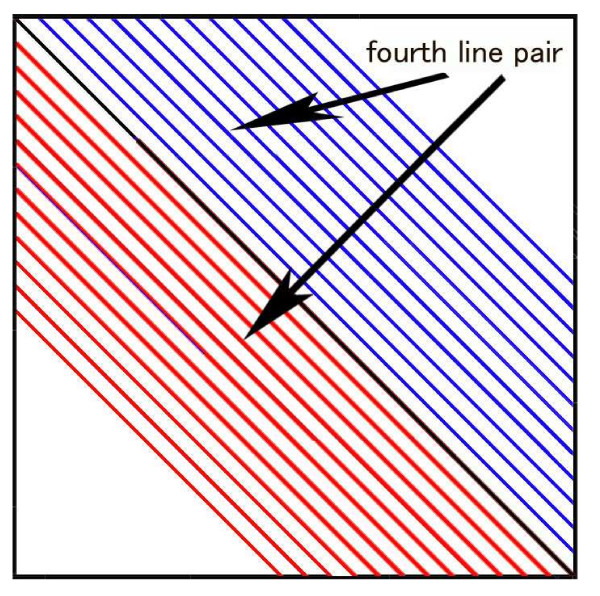
Sketch map of the 12 paired diagonals for calculating total skew correlation. See text for explanation. The arrows show the fourth line pair as an example.

As shown in Figure 5, where k means the line from 1 to 12 in turn and *β *is the separation between the diagonal lines. Each line can be considered as a vector of base skew, the blue lines for AT skews and red lines for CG skews. The 12 pairs of symmetrical lines have 12 correlation numbers *C*_*i*_, Positive values of *C*_*i *_mean positively correlated AT and CG skews, negative values mean negative correlation. The average of *C*_*i *_is defined as constant number correlation level (CNCL) of the target sequence.

CNCLβspecies−chr*=112∑i=112Ci
 MathType@MTEF@5@5@+=feaafiart1ev1aaatCvAUfKttLearuWrP9MDH5MBPbIqV92AaeXatLxBI9gBaebbnrfifHhDYfgasaacPC6xNi=xI8qiVKYPFjYdHaVhbbf9v8qqaqFr0xc9vqFj0dXdbba91qpepeI8k8fiI+fsY=rqGqVepae9pg0db9vqaiVgFr0xfr=xfr=xc9adbaqaaeGacaGaaiaabeqaaeqabiWaaaGcbaGaem4qamKaemOta4Kaem4qamKaemitaW0aa0baaSqaaGGaciab=j7aIbqaaiabdohaZjabdchaWjabdwgaLjabdogaJjabdMgaPjabdwgaLjabdohaZjabgkHiTiabdogaJjabdIgaOjabdkhaYjabcQcaQaaakiabg2da9KqbaoaalaaabaGaeGymaedabaGaeGymaeJaeGOmaidaaOWaaabCaeaacqWGdbWqdaWgaaWcbaGaemyAaKgabeaaaeaacqWGPbqAcqGH9aqpcqaIXaqmaeaacqaIXaqmcqaIYaGma0GaeyyeIuoaaaa@5059@

species-chr* means for which DNA sequence and *β *means the step size from one line to next.

### The definition of constant window correlation level of genome sequence

CNCL mentioned above is a correlation score of the BSDT of genomes. However, we know that for different species and different chromosomes, the genome sequences are quite different in total length. So the window length will be different, even though we can compare the CNCLs by choosing different parameter *β *values. For standardized comparison of chromosomes of different sizes, we define a new constant window correlation level (CWCL), where, compared with CNCL in last section, factor 1024 is replaced by

Window-number = genome-length/window-length

So CNCL is the absolute scale (1/1024 fraction of chromosome) correlation level of BSDT images and CWCL is the standardized scale correlation level. The arithmetic in CWCL is as for CNCL, but m, n < Window-number (not be 1024). CWCL is denoted as CWCLβspecies−chr*
 MathType@MTEF@5@5@+=feaafiart1ev1aaatCvAUfKttLearuWrP9MDH5MBPbIqV92AaeXatLxBI9gBaebbnrfifHhDYfgasaacPC6xNi=xH8viVGI8Gi=hEeeu0xXdbba9frFj0xb9qqpG0dXdb9aspeI8k8fiI+fsY=rqGqVepae9pg0db9vqaiVgFr0xfr=xfr=xc9adbaqaaeGacaGaaiaabeqaaeqabiWaaaGcbaGaem4qamKaem4vaCLaem4qamKaemitaW0aa0baaSqaaGGaciab=j7aIbqaaiabdohaZjabdchaWjabdwgaLjabdogaJjabdMgaPjabdwgaLjabdohaZjabgkHiTiabdogaJjabdIgaOjabdkhaYjabcQcaQaaaaaa@4193@, species-chr* and *β *has the same meaning as in equations (5) for CNCL.

## Materials

The eukaryotic genomic data was sampled as shown in Table 1. Full chromosome sequences were used, except in turkey and zebrafinch, for which pooled scaffolds of 100–300 kb downloaded from NCBI were used in the absence of full genome data.

## Abbreviations

Base Skew Double Triangle is abbreviated as BSDT, CNCL means Constant Number Correlation Level, CWCL is abbreviation of Constant Window Correlation Level, and WL is window length in this paper.

## Authors' contributions

XgD invented the visualization method, carried out calculation of parameters and drafted the manuscript. IH participated in resourcing and exploring the data, statistical analysis, literature review and modification of the manuscript. XmD conceived of the study, and participated in its design and writing. All authors read and approved the final manuscript.
